# An Enhancement in the Magnetocaloric Effect in a Composite Powder Based on Lanthanum Manganites

**DOI:** 10.3390/ma18214869

**Published:** 2025-10-24

**Authors:** Fidel Ivan Reyes Patricio, Cristhian Antonio Taboada Moreno, Ana María Bolarín Miró, Claudia Alicia Cortés Escobedo, María Isabel Reyes Valderrama, Félix Sánchez De Jesús

**Affiliations:** 1 Área Académica de Ciencias de la Tierra y Materiales, Universidad Autónoma del Estado de Hidalgo, Mineral de la Reforma 42184, Mexico; re295437@uaeh.edu.mx (F.I.R.P.); ta271740@uaeh.edu.mx (C.A.T.M.); abolarin@uaeh.edu.mx (A.M.B.M.); profe_5490@uaeh.edu.mx (M.I.R.V.); 2 Centro de Investigación e Innovación Tecnológica, Instituto Politécnico Nacional, Ciudad de México 02250, Mexico; ccortese@ipn.mx

**Keywords:** Magnetic refrigeration, composite, magnetocaloric effect, magnetic entropy change (ΔS_M_), relative cooling power (RCP), manganites, high-energy ball milling

## Abstract

This study presents a dual-phase lanthanum manganite ceramic composite based on a mixture of equal weight ratios of La_0.7_Ca_0.2_Sr_0.1_MnO_3_ and La_0.7_Ca_0.25_Sr_0.05_MnO_3_ designed to enhance the magnetocaloric effect (MCE) of individual compounds, under a low magnetic field (≤18 kOe). X-ray diffraction (XRD) analysis revealed the coexistence of two orthorhombic manganite phases corresponding to the individual compounds, with no secondary phases detected. Temperature-dependent magnetization measurements in the composite evidenced two Curie temperatures at 286.8 K and 307.6 K, reflecting the effect of Ca^2+^ and Sr^2+^ concentrations. Arrott plots and β parameters confirmed that the phase transition is of second order. Although the maximum magnetic entropy change (ΔS_M_) of the composite is slightly lower than that of the individual manganite phases, its relative cooling power (RCP) reaches 188.82 J·kg^−1^, with an extended operational temperature window (OTW) of approximately 85 K, spanning from around 243 K to 328 K. This broad OTW enables efficient operation over a wider temperature range compared to similar materials, such as the individual La_0.7_Ca_0.2_Sr_0.1_MnO_3_ and La_0.7_Ca_0.25_Sr_0.05_MnO_3_ compounds, which exhibit an RCP of 55.24 and 65.12 J·kg^−1^, respectively, under a comparable magnetic field (~18 kOe). The improved magnetocaloric performance is attributed to interfacial exchange coupling and strain-mediated effects that broaden the ΔS_M_ response and generate a non-additive RCP. These results demonstrate that interphase coupling and microstructural tuning effectively broaden the operating temperature range for magnetic refrigeration under moderate fields, making this composite a strong candidate for practical cooling applications.

## 1. Introduction

Magnetic refrigeration, exploiting the magnetocaloric effect (MCE), has attracted significant attention as a sustainable and efficient alternative to conventional gas compression cooling technologies. The MCE refers to the reversible temperature change in a material upon the application or removal of an external magnetic field under adiabatic conditions, enabling energy-efficient thermal management without environmentally noxious refrigerants [1,2,3,4]. Among magnetocaloric materials, gadolinium (Gd)-based alloys are considered the most important systems, exhibiting large magnetic entropy changes (ΔS_M_) near room temperature due to their strong magnetic moment and favorable magnetic transitions. However, the practical deployment of Gd-based materials faces obstacles, including high production costs, complex synthesis routes, and long-term stability issues [3]. In contrast, ceramic magnetocaloric materials, particularly manganites of the LaMnO_3_ family, present cost-effective and chemically stable alternatives with tunable magnetic properties achieved via substitutional doping [4,5,6,7,8]. The partial replacement of La^3+^ by alkaline earth metals such as Ca^2+^ or Sr^2+^ adjusts critical parameters including the Curie temperature (Tc), thereby optimizing the MCE near ambient temperatures [4]. These dopants modify the Mn^3+^/Mn^4+^ ratio, enhancing the double-exchange (DE) interactions responsible for ferromagnetic ordering. Such modifications promote ferrimagnetic states through structural distortions involving MnO_6_ octahedra and Jahn–Teller effects, which affect the magnetic entropy change and widen the operating temperature window (OTW), resulting in increased relative cooling power (RCP) [5,6,7,8,9,10,11,12,13,14,15,16,17]. Nevertheless, despite these advantages, manganites generally exhibit lower maximum entropy changes and relatively narrow temperature spans when compared to Gd-based materials, which constrains their overall refrigeration efficiency and limits their practical applications [6,9,10,11,17].

To overcome these limitations shown by doped lanthanum manganites, considerable research has been directed toward designing composite and mixed-phase manganite systems. Such strategies aim to broaden the OTW and enhance the RCP by combining phases with different Curie temperatures. Composite-based manganite has been explored to induce multiple magnetic transitions, thus expanding the temperature span of the magnetocaloric effect. In this context, Maalam et al. [8] produced (La_0.45_Nd_0.25_)Sr_0.3_MnO_3_/5Cu composites via solid-state reactions; analysis using scanning electron microscopy (SEM) revealed CuO phases located predominantly at the grain boundaries, which influenced magnetic interactions and an improvement in the magnetocaloric properties. Notably, these composites demonstrated a decrease in Tc but a more than twofold increase in the maximum ΔS_M_ compared to parent compounds, highlighting enhanced magnetocaloric performance. Additionally, Zhou et al. [9] explored copper doping effects in (1 − x)La_0.67_Sr_0.33_MnO_3_/xCu composite coatings, finding significant impacts on the structural, electrical, and low-field magnetoresistance properties that are relevant for tuning the magnetocaloric response. Reported ΔS_M_ peak values in manganite composites generally range between 3 and 5 J·kg^−1^·K^−1^ under magnetic field changes of 1 to 5 T, with relative cooling power (RCP) values often broadened over wider working temperature intervals due to the composite nature. Focused on the use of manganites as individual compounds, Jeddi et al. [10] reported an RCP of 210 J·kg^−1^· and |ΔS_M_| of 0.9 J·kg^−1^·K^−1^ at 3 T with a Tc increase from 255 to 365 K using a 40:60 weight ratio of La_0.6_Ca_0.4_MnO_3_ and La_0.6_Sr_0.4_MnO_3_, extending the work temperature range. Other studies such as those by Sellami et al. [11], Akça et al. [12], M’nassri et al. [13], and Yigiter et al. [14] reported ΔS_M_ values ranging from 1.8 to 4.1 J·kg^−1^·K^−1^ and an RCP up to 303 J·kg^−1^· under applied magnetic fields of 5 T for composites of La_0.7_Ca_0.3_MnO_3_ and La_0.7_Ag_0.3_MnO_3_.

Additional approaches include adjusting the proportion of rare-earth and transition metal ions and performing post-synthesis treatments such as thermal annealing to improve phase diffusion and entropy stability. For instance, Bisht et al. [15], Kılıç et al. [16], and Boubekri et al. [17] have shown a wide range of operating temperatures with stable magnetocaloric responses using optimized ceramic mixtures and simulations. Bouhani et al. [18] emphasized that maximizing ΔS_M_ is critical for material sensitivity, while RCP determines the cooling capacity of the material. Therefore, materials with a large RCP and a large entropy change are ideal for improving cooling efficiency.

Focused on the use of manganite for obtaining composites, Ezaami et al. [19] synthesized composites formed by a mixture of La_0.7_Ca_0.25_Sr_0.05_MnO_3_ and La_0.7_Ca_0.2_Sr_0.1_MnO_3_ compounds using the solid-state reaction method, where the Curie temperature of the composites was found to be intermediate between those of the parent compounds, exhibiting a double transition indicative of two different values. The ΔS_M_ values of these composites vary accordingly between those of the parents, with optimized ΔS_M_ maxima achieved in certain compositions [20,21,22,23,24,25,26,27,28,29,30,31]. These results show a promising way to widen the OTW to near room temperature and, at the same time, increase the RCP.

Several studies have shown that annealing or thermal treatment significantly improves the magnetocaloric properties of manganite-based composites due to the promotion of the diffusion process forming intermediate phases. For instance, in the work by El Amrani et al. [23], Nd_0.7_Sr_0.3_MnO_3_:CuO composites were synthesized and heat-treated at 1473 K, leading to enhanced magnetic entropy change (|ΔS_M_| ≈ 5.14 J·kg^−1^·K^−1^ at 5 T) and an improved relative cooling power (RCP ≈ 232 J·kg^−1^) compared to the parent manganite. Similarly, Giri et al. [24] investigated La_0.7_Ca_0.3_MnO_3_-MO (MO = CuO, CoO, NiO) composites and demonstrated that the addition of these metal oxides affected the ferromagnetic transition temperature and enhanced the RCP values under applied fields, attributing part of the improvement to changes induced by thermal processing. Moreover, Yilmaz et al. [25] designed a composite consisting of La_0.67_Ca_0.27_Sr_0.06_MnO_3_ and La_0.7_Sr_0.3_Mn_0.94_Ni_0.06_O_3_, finding that thermal treatment and appropriate phase blending yielded a broadened magnetocaloric effect due to the extended Curie temperature of the composite, with RCP values up to 317.7 J·kg^−1^ under 5 T. Similarly, Fkhar et al. [27] applied heat treatment at 1473 K for 12 h to La_0.45_Nd_0.25_Sr_0.3_MnO_3_/CuO composites, which resulted in the diffusion of both compounds, enhancing some magnetocaloric properties of the composite compared with individual compounds. These examples highlight the widespread application of post-heat treatments in manganite-based composites and emphasize the importance of controlling phase coexistence for optimizing material properties.

Therefore, this work focuses on the study of a composite powder obtained from a mixture of two lanthanum-based manganites, both doped at site A. Each individual phase exhibits a magnetocaloric effect (MCE) within a narrow temperature range, defined by its respective Curie temperature. However, for practical applications in magnetic cooling, especially at room temperature, it is essential to have a longer operating temperature range. To overcome this limitation, the present study explores a controlled 50:50 mixture of both manganite phases, with the main objective of enhancing the magnetocaloric effect by extending the effective OTW. This approach seeks to improve the RCP without the need to maximize ΔS_M_. Although the maximum value of ΔS_M_ may remain comparable or even slightly reduced relative to that of the pure phases, the overlap of the entropy change peaks of both manganites results in a broader MCE peak, leading to a significant improvement in RCP. On the other hand, the synthesis of manganites is carried out by high-energy ball milling, a technique known to induce structural distortions and generate beneficial crystallographic defects that can enhance the magnetocaloric response. Compared to conventional wet chemical methods, this mechanochemical route offers notable advantages in terms of scalability, cost-effectiveness, and process simplicity while maintaining a high degree of reproducibility.

As aforementioned, the aim of this work is to investigate the synergistic effect of two lanthanum manganites as a composite powder on the OTW, ΔS_M_, and the RCP in order to enhance their MC performance and also provide a deeper understanding of the physical mechanisms governing RCP improvement. Consequently, the results of this work contribute to the rational design of functional magnetocaloric materials for solid-state cooling technologies, emphasizing the fundamental role of synthesis techniques in adapting the properties of advanced materials. This study is focused on demonstrating that the chosen synthesis approach can enhance the magnetocaloric performance of manganite composites under practical, low-field conditions (18 kOe), making them suitable for domestic applications.

## 2. Materials and Methods

### 2.1. The Synthesis of Manganites and Preparation of the Composite

High-purity La_2_O_3_, SrO, CaO, and Mn_2_O_3_ powders, all from Sigma-Aldrich (St. Louis, MO, USA) with purities ranging from 99.8% to 99.9%, were used as precursors for the synthesis of individual manganites, La_0.7_Ca_0.25_Sr_0.05_MnO_3_ (LCSM0.05) and La_0.7_Ca_0.2_Sr_0.1_MnO_3_ (LCSM0.1), according to the following reaction:

(1) $$0.7{\mathrm{La}}_{2}O_{3\;}+(0.3-x)\mathrm{CaO}+\;\mathrm{xSrO}\;+\;{\mathrm{Mn}}_{2}O_{3\;}+\;1/2O_{2\;}\to \;{2\mathrm{La}}_{0.7}{\mathrm{Ca}}_{0.3-x}{\mathrm{Sr}}_{x}{\mathrm{MnO}}_{3}$$

**Figure 1:**
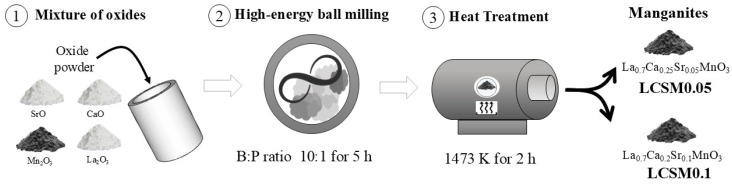
A schematic representation of the experimental procedure used to obtain single manganites (LCSM0.05 and LCSM0.1).

For the synthesis of the individual manganites, 5 g of the stoichiometric oxide’s mixtures, according to Equation (1), was introduced, together with 50 g of hardened steel balls of 12.7 mm in diameter, into a 60 mL hardened steel vial (6 × 10^−5^ m^3^) and was subjected to a high-energy ball milling process in an air atmosphere, using a Mixer/Mill (SPEX model 8000D, Metuchen, NJ, USA), for 5 h at a ball-to-powder weight ratio of 10:1 for 5 h. The milled powders were subsequently annealed in an air atmosphere at 1473 K with a heating rate of 10 °C/min and held for 2 h and air-cooled using a tubular muffle furnace (Lindberg/Blue M model STF54459C, Riverside, MI, USA) to achieve complete crystalline phase formation, in agreement with previous studies [20]. A schematic representation of the experimental process is presented in Figure 1.

**Figure 2:**
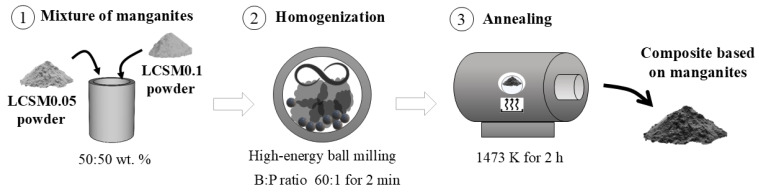
A schematic representation of the experimental procedure used to prepare the composite based on the synthesized manganites: LCSM0.05 and LCSM0.1.

To prepare the composite, based on equal weights (50 wt. % each) of the manganites synthesized previously, LCSM0.05 and LCSM0.1, the mixture of manganites in proper proportions was attained, as shown in Figure 2. A total of 2 g of the mixture with 12 g of a hardened steel ball of 9.525 mm in diameter, using a ball-to-powder weight ratio of 60:1, was milled in the high-energy ball miller for 2 min in order to homogenize the mixture, using methanol as a process control agent, only during this step, to prevent agglomeration and ensure a uniform dispersion without modifying the stoichiometry of the pre-synthesized manganites [20]. The obtained powder was then annealed at 1473 K for 2 h as a strategy to promote particle bonding, as some studies have demonstrated the benefit of annealing in terms of the magnetocaloric properties of composites [21,22,23,24,25,26,27,32,33,34,35,36,37,38,39,40,41]. A flowchart of the experimental procedure is shown in Figure 2.

### 2.2. Crystal Structure and Microstructure

The crystal structure of the synthesized manganites was analyzed using a Bruker D8 Advance X-ray diffractometer (Bruker D8 Advance, Karlsruhe, Germany) with Cu Kα radiation (λ = 1.541874 Å). Rietveld refinement was performed with Material Analysis Using Diffraction (MAUD) software Version 2.992, while structural identification was assisted by the use of the Inorganic Crystal Structure Database (ICSD). Diffraction peak profiles were modeled using a pseudo-Voigt function, combining Gaussian and Lorentzian components. The Gaussian term accounts mainly for instrumental and microstrain broadening, while the Lorentzian reflects size effects. The instrumental resolution function was calibrated with a standard SiO_2_ pattern (CIF COD 1010921), and zero-shift corrections were applied, estimating a detection limit of 2.5 wt. % Lattice parameters, phase fractions, crystallite size, and microstrain were refined simultaneously in MAUD. The uncertainties of the structural parameters were derived from the covariance matrix, considering statistical propagation and parameter correlations. Additionally, crystal structure simulations were performed using Visualization for Electronic and Structural Analysis (VESTA) software Version 4.6.0. The morphology and particle size of the composite powders were examined by scanning electron microscopy (SEM, HITACHI model TM3030, Ibaraki, Japan) equipped with an energy-dispersive spectroscopy system (EDS, QUANTAX model 75, Bruker Nano GmbH, Berlin, Germany) for chemical analysis.

### 2.3. Magnetic and Magnetocaloric Properties

Magnetic and magnetocaloric measurements were performed using a VSM vibrating sample magnetometer (MicroSense EV7, Lowell, MA, USA); this piece of equipment has an instrumental uncertainty of 0.005 Oe in magnetic field measurement. Isothermal magnetization curves (M vs. H) were obtained in the temperature range covering the ferromagnetic-to-paramagnetic transition (±39 K around T_C_, with ΔT = 3 K), under applied magnetic fields from 0 to 18 kOe after zero-field cooling (ZFC). In this test, magnetization was measured in a three-step protocol: 0–1 kOe at 100 Oe intervals, 1–5 kOe at 600 Oe intervals, and 5–18 kOe at 1 kOe intervals. The Curie temperature (T_C_) was determined from temperature-dependent magnetization measurements under an applied field of 10 kOe around the Curie temperature of each component. Arrott plots were created and ΔS_M_ and RCP were calculated from isothermal magnetic curves. Electron spin resonance (ESR) spectra were obtained in a Magnettech Miniscope MS 400 (MT MagnetTech GmbH, Berlin, Germany) spectrometer in the X-band (9.4 GHz), equipped with a JUMO dTRON304 temperature system (JUMO GmbH & Co. KG, Fulda, Germany).

The uncertainty in the −∆S_M_ calculations was estimated by the error propagation of the discrete Maxwell relation, considering the instrumental error in magnetization (δM = ±0.005 emu), the temperature step (ΔT = 3 K), the maximum applied field (ΔH = 1.8 T), and the sample mass (~0.2208 g). The resulting deviation is approximately ± 0.0192 J Kg^−1^ K^−1^. This uncertainty, which mainly arises from the temperature step and magnetization precision, was taken into account in the ∆S_M_ analysis and is representative of the experimental confidence range.

## 3. Results and Discussion

### 3.1. Crystal Structure

**Figure 3:**
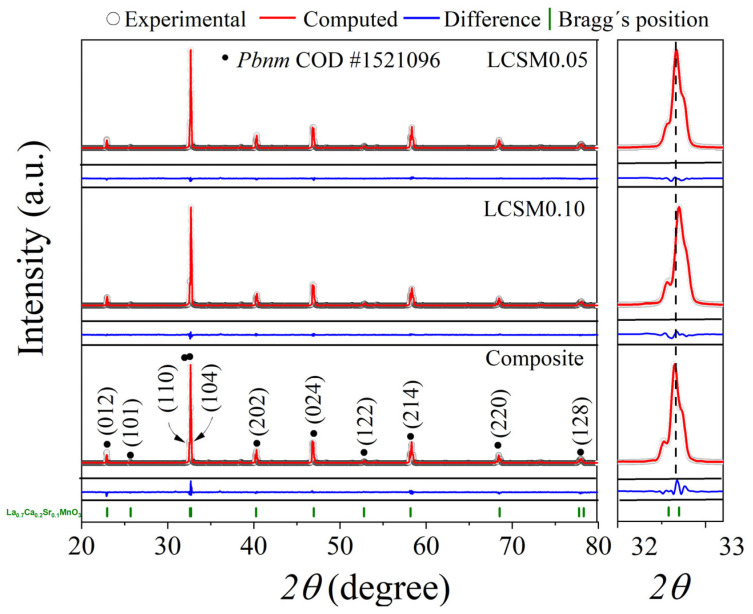
The X-ray diffraction (XRD) patterns of the individually synthesized manganites (LCSM0.05, LCSM0.1) and the composite.

Figure 3 shows the X-ray diffraction (XRD) patterns of the individually synthesized manganites (LCSM0.05 and LCSM0.1) and the composite. The single-phase manganites show diffraction peaks corresponding to the orthorhombic perovskite structure of calcium-doped lanthanum manganite (La_0.65_Ca_0.325_MnO_2.975_, COD 1521096, *Pbnm*). Slight shifts in the 2θ positions of certain diffraction peaks, particularly those corresponding to the (110) and (104) planes (shown in detail on the right side of Figure 3), correspond to a change in interplanar distances, depending on strontium content, as larger strontium ions (ionic radius 1.26 Å) substitute smaller calcium ions (ionic radius 1.12 Å). The XRD pattern on the composite displays the same set of diffraction peaks, corresponding to a mixture of both the orthorhombic manganites.

The main diffraction peak exhibits a shift toward lower 2θ angles, indicating an expansion of the unit cell. This behavior can be attributed to the incorporation of ions with larger ionic radii, which induce lattice strain during the sintering process, while the crystal structure is preserved. The XRD patterns confirm the successful synthesis of the compounds and the obtaining of the composite based on the individual manganites.

To quantify the phases and modifications of the orthorhombic structure, Rietveld refinement was performed on the XRD patterns (Figure 3). The refinement results, summarized in Table 1, confirm that the single compounds (LCSM0.05 and LCSM0.1) crystallize in a single orthorhombic phase, while the composite consists of a mixture of both manganite phases. The comparison between the experimental data and the cell parameters of the reference XRD pattern suggests that the presence of Ca^2+^ and Sr^2+^ in the manganite crystal structure does not significantly alter the original crystal structure. Rietveld refinement determined that the weight percentage of manganites in the composite powder was 50.4 and 49.6% of LCSM0.05 and LCSM0.1, respectively.

Table 1 also includes crystallite size, which is similar for the LCSM0.1 and LCSM0.05 samples, while in the case of the composite, the crystallite size of both phases is slightly larger compared to the precursor manganites, because of the heat treatment that causes recrystallization processes. Table 1 presents the refinement quality evaluated through the R_wp_ and χ^2^ parameters. Although the R_wp_ values deviate from the conventional standards, likely due to experimental data quality, magnetic contributions, and background effects, the χ^2^ values indicate a satisfactory agreement between observed and calculated profiles. Given that χ^2^ = (Rwp/Rexp)^2^, this parameter provides a more reliable assessment of refinement quality, as it accounts for data statistics, background subtraction, and the consistency between experimental and simulated patterns [42].

Although the refinement yielded an adequate goodness of fit, it is plausible that minor interfacial phases formed during annealing as a result of limited Sr–Ca interdiffusion between the two manganite components. These regions, with slightly shifted stoichiometry and Mn^3+/^Mn^4+^ ratios, could modify local exchange interactions and contribute to the broadening of the magnetic transition. Although their fraction is likely below the XRD detection limit and cannot be quantified by Rietveld refinement, their presence may be inferred from subtle features in temperature-dependent magnetization and ESR response, both sensitive to local structural and magnetic inhomogeneities.

Due to the effect of the Mn^3+^/Mn^4+^ ratio on double-exchange magnetic interactions in manganites, an analysis of the distortion of Mn-O-Mn bond octahedra was carried out, evaluating the angles. The different oxidation state of Mn modifies the magnetic interactions and magnetocaloric properties of the composite powder, underscoring the importance of structural modifications to optimize the performance of manganite based-materials.

The bond angles between the Mn and O ions are presented in Table 2. Specifically, Mn_1_-O-Mn_1_ represents the angles in the b-direction, while Mn_2_-O-Mn_2_ corresponds to the angle in the c-direction. The data show a slight structural distortion in the composite compared to the former LCSM0.05 and LCSM0.1, a phenomenon attributed to Sr^2+^ diffusion between single compounds during annealing.

Manganites possess a perovskite-type structure whose distortion arises from the ionic radius mismatch between A-site dopants (La, Sr, Ca) and the host lattice and from oxygen vacancies induced by doping. These distortions, strongly influenced by the Jahn–Teller effect, modify the Mn^3+^–Mn^4+^ ratio, affecting the Curie temperature and the magnetic interactions. While the Mn^3+^–Mn^4+^ interaction via the double-exchange mechanism promotes ferromagnetism, the prevalence of Mn^3+^ favors antiferromagnetism; the variation in bond lengths and angles due to doping thus tunes the magnetic and magnetocaloric properties of the material [23]. In mixed-valence perovskite manganites, magnetic exchange (double-exchange and super-exchange) and therefore the Curie temperature [20] are highly sensitive to the tilt of the MnO_6_ octahedron and dependent on the Mn-O-Mn geometry because the 3d–2p orbital overlap (bandwidth) depends strongly on the Mn-O-Mn angle; therefore, modest modifications in the bond angle change the effective hopping amplitude and can thus alter magnetic coupling. In the studied material, formed by a mixture of two manganites with very similar compositions, differences of a few degrees in the Mn-O-Mn bond angle are expected, as they produce important changes in magnetic properties, allowing us to modulate the magnetic properties, such as Curie temperature.

### 3.2. Particle Morphology and Chemical Composition

**Figure 4:**
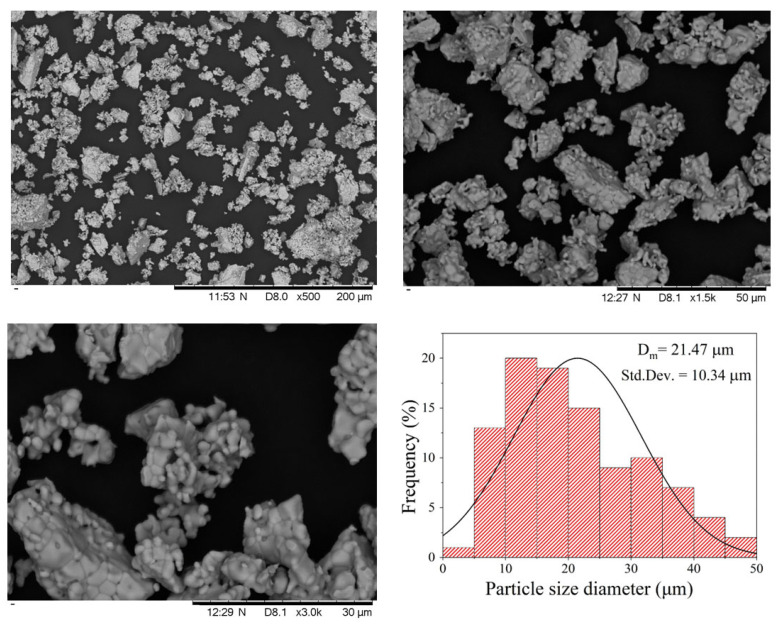
Representative SEM micrographs of the composite, showing particle morphology and particle size distribution.

To analyze the microstructural characteristics of the composite powder, including particle morphology and size distribution, scanning electron microscopy (SEM) was employed. Representative micrographs at different magnifications are shown in Figure 4.

As can be seen in Figure 4, the composite powder shows a broad monomodal asymmetric particle size distribution (5–45 μm) with media of 21.47 μm, with a relatively wide standard deviation (3.14 μm) arising from particle agglomeration. The presence of a monomodal distribution, despite consisting of two manganites, is explained by particle agglomeration during milling and annealing, which masks any separation of individual size populations. Most particles appear to originate from the coalescence of finer grains, leading to polyhedral morphologies that suggest an incipient sintering process. This coalescence, particularly between LCSM0.05 and LCSM0.1 particles, not only promotes the mechanical consolidation of the composite but also enhances interfacial connectivity between crystallites. Such microstructural features play a decisive role in the MCE, since the density of grain boundaries and the degree of chemical homogeneity modulate the double-exchange interactions and spin alignment. Consequently, the optimized microstructure in the composite powder strengthens the cooperative magnetic response, thereby improving the magnitude and reversibility of the magnetic entropy change.

**Figure 5:**
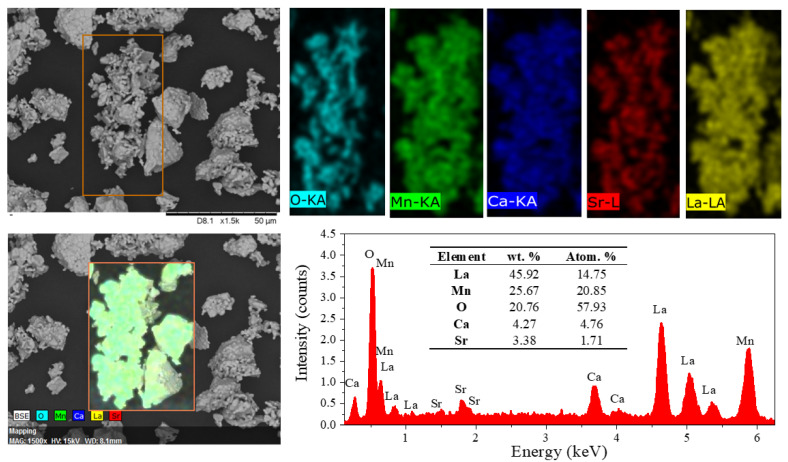
SEM micrographs and compositional analysis of composite. Top: Elemental maps of O, Mn, Ca, Sr, and La, evidencing homogeneous spatial distribution. Bottom left: BSE image showing particle morphology. Bottom right: EDS spectrum confirming intended stoichiometry of composite.

Energy-dispersive X-ray spectroscopy (EDS) was employed to qualitatively evaluate the chemical composition and elemental distribution across the composite powder, as shown in Figure 5. The corresponding spectra display only the expected peaks of the constituent elements, without detectable impurities, and the quantified values are consistent with the compositions of the manganites forming the composite. Additionally, elemental analysis is congruent with the presence of a mixture of both manganites. While the overall distribution of elements is homogeneous, variations in strontium content are observed in certain particles, confirming that individual particles have different compositions, consistent with the coexistence of manganites with distinct stoichiometries within the composite.

### 3.3. Magnetic Properties

**Figure 6:**
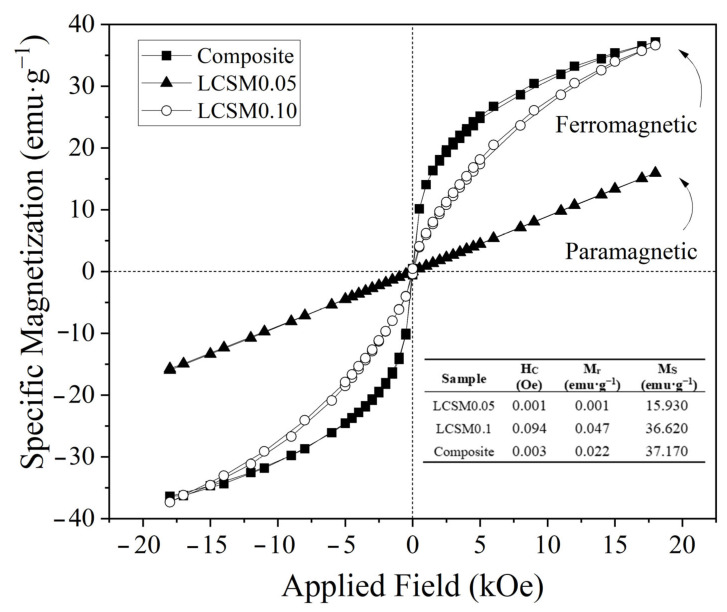
Magnetic hysteresis loops at room temperature (293 K) for LCSM0.05, LCSM0.1, and the composite. In the inset, the magnetic parameter, coercivity (Hc), remanent magnetization (Mr), and specific magnetization at 18 kOe (Ms) are presented.

As is well known, magnetic properties are highly sensitive and critically dependent on composition and crystal structure, as previously discussed in the structural characterization. Previous results indicate that the presence of manganites with slight compositional variations in the composite powder can influence its magnetic behavior and, consequently, its magnetocaloric performance. In this study, it is anticipated that the coexistence of different manganites within the composite powder could enhance the magnetocaloric response, particularly the RCP. To test this hypothesis, magnetic hysteresis loops at room temperature (298 K) were measured for the composite and the individual compounds, and the results are shown in Figure 6. As observed in the table included as an inset in Figure 6, the pure compound LCSM0.05 exhibits paramagnetic behavior with a specific magnetization of 15.93 emu·g^−1^ at 18 kOe, whereas LCSM0.1 shows ferromagnetic ordering with a specific magnetization of 36.62 emu·g^−1^, highlighting the impact of small amounts of Sr^2+^ on the magnetic properties of the manganite. These results are consistent with previously reported studies [2,3,4,5,6,7,8,9,10,11] and in agreement with the XRD refinement parameters listed in Table 1 and Table 2, where Sr doping in LCSM0.1 induces higher structural distortion, favoring double-exchange interactions at room temperature. In Figure 6, it is also observed that the composite exhibits a slim magnetic hysteresis loop (coercive field ~11.57 Oe) with ferromagnetic ordering and a specific magnetization of 37.17 emu·g^−1^ at 18 kOe, making it an excellent soft magnetic composite powder for magnetocaloric applications, since less energy is required for cyclic magnetization and demagnetization. The magnetic response of the composite thus reflects the mass-weighted contribution of its individual constituents, confirming that its overall behavior arises from the combined properties of the two manganites [27,28,29,30,31].

**Figure 7:**
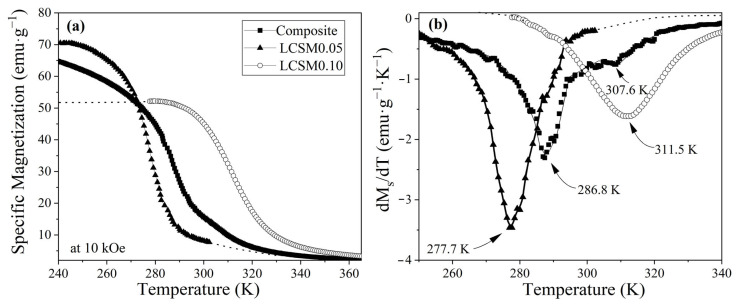
(**a**) Specific magnetization curves (Ms) versus temperature under applied field of 10 kOe and (**b**) dM/dT of specific magnetization curves of LCSM0.05, LCSM0.1, and composite.

Moreover, the temperature dependence of the specific magnetization, shown in Figure 7, was used to determine the Curie temperature (T_C_) by analyzing the derivative of the magnetization curve. T_C_ was identified at the maximum of the first derivative (dM/dT), which corresponds to the inflection point marking the transition from the ferromagnetic to the paramagnetic state. Measurements were carried out under a constant magnetic field of 10 kOe, and the obtained values were compared with those reported in the literature and presented in Table 3. As displayed in Figure 7a, the Curie temperature was determined as 277.7 K for LCSM0.05 and 311.5 K for LCSM0.1, with both values being in agreement with those previously reported for analogous manganite systems [30]. In contrast, the composite exhibits two distinct peaks at 286.8 K and 307.6 K, reflecting the coexistence and interaction of ferromagnetic and paramagnetic phases within the material. Additionally, the curve of the composite presents a very small deviation, indicating intermediate states that arise from the phase interaction. This behavior corroborates the presence of two predominant phases in the composite while simultaneously generating intermediate responses, thereby confirming the dual-phase nature of the system. Such coexistence is particularly advantageous for applications requiring tunable magnetic properties, such as magnetic refrigeration. Furthermore, the persistence of two main transitions together with the intermediate features after thermal treatment demonstrates that the designed composite preserves the targeted multiphase structure, enabling enhanced performance through the synergistic contribution of the different phases.

The dual Curie temperatures observed in the composite sample can be quantitatively explained by the nearly equal phase fractions determined by Rietveld refinement (50.4% LCSM0.05 and 49.6% LCSM0.1). The lower T_C_ transition corresponds to the LCSM0.05 phase, while the higher one corresponds to LCSM0.1. The comparable weight fractions of both phases justify the presence of two well-defined peaks of similar magnitude (286.8 and 307.6 K) arising from their interactions. Thus, the overall ΔS_M_ response results from the additive contributions of both phases, broadening the operational temperature window between 243 and 328 K and yielding an enhanced RCP of 188.82 J·kg^−1^, which increased by almost threefold compared with the single phases.

Although the studied composite is formed by two different lanthanum manganites with a very similar composition, their Curie temperatures are different, with a difference of ≈33.8 K between the two components, at 277.7 K and 311.5 for LCSM0.05 and LCSM0.1, respectively, as shown in Figure 7b, which correlates with the subtle but reproducible bond angle changes and with the complementary magnetic tests presented previously.

**Figure 8:**
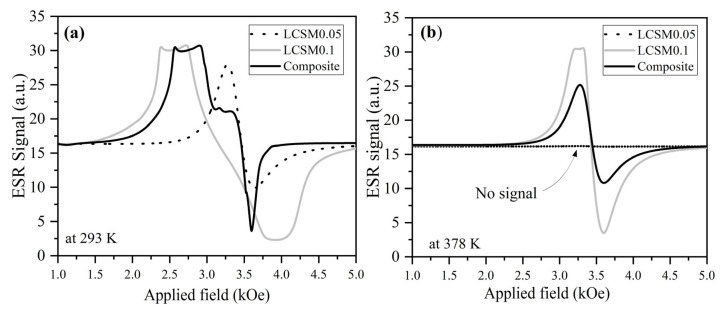
Electron spin resonance (ESR) spectra of LCSM0.05, LCSM0.1, and composite measured at (**a**) 293 K and (**b**) 378 K.

To further corroborate our findings, we conducted electron spin resonance (ESR) analysis, a technique highly sensitive to spin dynamics and local magnetic correlations, which can reveal distinct signatures for each manganite phase and their composite. As shown in Figure 8, the ESR spectra recorded at 293 K and 378 K for the individual compounds and the composite exhibit notable differences. Both LCSM0.05 and the composite display a characteristic hump in the signal, a hallmark of the coexistence of ferromagnetic (FM) and paramagnetic (PM) regions, as commonly reported in phase-separated manganites [43]. The values of the g factor were 1.992, 1.988, and 2.179 at 293 K for LCSM0.05, LCSM0.1, and the composite, respectively. In contrast, g achieves values of 1.983, 1.998, and 2.010 for LCSM0.05, LCSM0.1, and the composite at 378 K, respectively. The value close to g ≈ 2 observed in LCSM0.05 is typical of a paramagnetic response above its Tc, while the deviation found in LCSM0.1 reflects stronger ferromagnetic exchange near its Tc. The composite shows the highest g values at both temperatures, indicating greater structural anisotropy. In this case, some Mn-O-Mn bonds present different angles and distances, and spin–orbit coupling is uneven. As a result, not all spins reach the ferromagnetic–paramagnetic transition simultaneously but progressively under slightly different conditions, which broadens the temperature dependence of ΔS_M_ and the RCP simultaneously. The effective *g* factor was determined using the following equation [44].

(2) $$g_{\mathrm{eff}}=\frac{h\nu }{\mu_{B}H_{\mathrm{res}}}$$

where *h* is Planck’s constant, u the microwave frequency, m_B_ the Bohr magneton, and *H_res_* the resonance magnetic field. Values of g close to 2 are typical of itinerant e_g_ electrons associated with Mn ions, reflecting the influence of the Mn^3+^/Mn^4+^ ratio and their exchange interactions. The slight decrease in *g* at 378 K indicates a transition to predominantly paramagnetic behavior as thermal fluctuations suppress long-range FM order.

### 3.4. Magnetocaloric Properties: ΔS, OTW, and RCP

**Figure 9:**
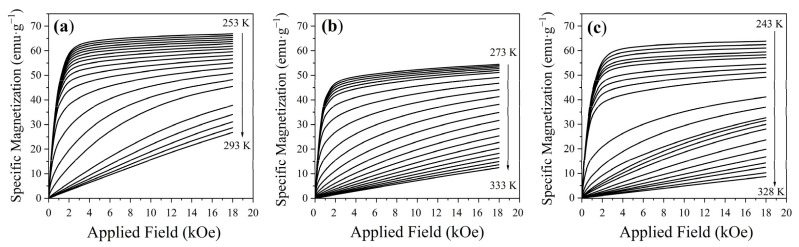
Isothermal magnetization curves M_s_(H, T) of (**a**) LCSM0.05, (**b**) LCSM0.1, and (**c**) composite.

Isothermal magnetization curves M_s_(H, T) were recorded at different temperature intervals, ranging from 243 K to 328 K, depending on the Curie temperature of each manganite sample, under an applied magnetic field from 0 to 18 kOe, to evaluate the magnetocaloric properties. Figure 9 shows the evolution from ferromagnetic to paramagnetic order in the three samples as the temperature increases. The widened OTW observed in the composite is attributed to the coexistence of both manganites, as previously evidenced in the magnetic hysteresis loops.

**Figure 10:**
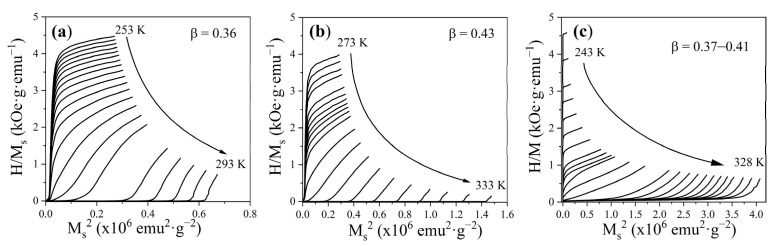
Arrott curves for (**a**) LCSM0.05, (**b**) LCSM0.1, and (**c**) composite.

To determine the order of the ferromagnetic-to-paramagnetic transition, Arrott plots were constructed, representing *H*/*M* as a function of *M*^2^, based on the isothermal magnetization data in Figure 10. This method, derived from Landau’s mean-field theory, relies on the Banerjee criterion [34], according to which positive slopes of the isothermal lines correspond to a second-order transition, while negative slopes indicate a first-order one. In addition, the Landau coefficient *β* was analyzed using the modified Arrott–Noakes method, based on the equation of state (H/M)^1/γ^ = α ε + bM^1/*β*^, where ε = (T − Tc)/Tc. From the high-field linear region of the Arrott plots (Figure 10), the spontaneous magnetization was extracted and fitted to M(T) ∝ (−ε)^β^, yielding β = 0.36 ± 0.02 (LCSM0.05), 0.43 ± 0.05 (LCSM0.1), and 0.37 ± 0.02–0.41 ± 0.12 (composite). The obtained critical parameter values together with the positive slopes throughout the investigated temperature range of the Arrott plots shown in Figure 10 confirm that all the samples exhibit a second-order nature of the transitions. This behavior is consistent with earlier reports on La-based manganites substituted with Ca and Sr [4,7,14,32,33], where substitution alters the Mn^3+^/Mn^4+^ ratio and the Mn-O-Mn bond angle but maintains the dominance of the double-exchange mechanism, typically giving rise to continuous transitions. In the LCSM0.05 and LCSM0.1 manganites, the smooth decrease in magnetization with temperature reflects the gradual suppression of ferromagnetic ordering, whereas in the composite, the coexistence of both parent phases preserves the same second-order character, without evidence of discontinuities or thermal hysteresis that would suggest a first-order process. The confirmation of second-order transitions in both the single-phase manganites and the composite is technologically relevant, since such transitions are associated with reversible entropy changes, broad operating temperature windows, and minimal hysteresis losses, features that are highly desirable for efficient magnetic refrigeration.

Δ*S*_M_, as a measurement of the magnetocaloric behavior of the materials, was calculated from the isothermal magnetization curves (Figure 9) using Maxwell’s relation [26]:

(3) $${\Delta S}_{M}(T,H)=-\mu_{0}\int_{0}^{H}{\left(\frac{dM}{dT}\right)}_{H}dH$$

where μ_0_ is the permeability of free space, M is magnetization, T is the absolute temperature, H is the applied magnetic field, and negative values denote the normal magnetocaloric effect. In practice, the integral was approximated via the trapezoidal rule and the derivative by central finite differences, yielding the following:

(4) $${|\Delta S}_{M}(T,H)|=\sum_{i}\frac{M_{i+1}\left(T_{i+1}\cdot H\right)-M_{i}(T_{i}\cdot H)}{T_{i+1}-T_{i}}\Delta H$$

where *M*_*i*_ denotes the experimentally measured magnetization at temperature T_*i*_ and field *H*.

**Figure 11:**
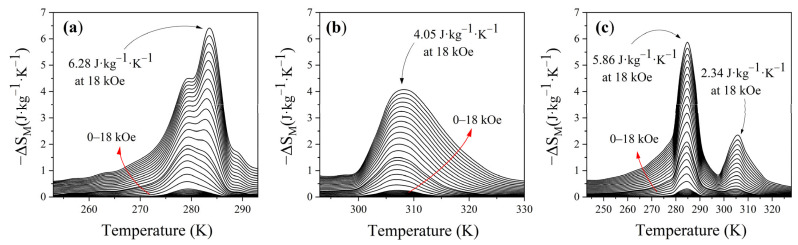
The temperature dependence of the entropy changes for (**a**) LCSM0.05, (**b**) LCSM0.1, and (**c**) the composite.

Plots of −Δ*S*_M_ versus temperature for the manganites LCSM0.05 and LCSM0.1 and the composite are shown in Figure 11. In each case, the maximum magnitude of Δ*S*_M_ occurs near the corresponding Curie temperature (T_C_), with there being two maximum magnetic entropy changes under an applied magnetic field of 18 kOe of 5.86 and 2.34 J·kg^−1^·K^−1^ for the composite and 4.05 and 6.28 J·kg^−1^·K^−1^ for LCSM0.1 and LCSM0.05, respectively, in line with the expectations for magnetocaloric materials, since ferromagnetic order progressively decreased above T_C_. Conversely, as can be observed in Figure 11, the Δ*S*_M_ versus T curve presented by the composite exhibits two well-resolved entropy maxima and a broad, nearly continuous Δ*S*_M_ response extending from ≈243 K to ≈328 K (≈85 K span) with local Δ*S*_M_ values remaining higher than 0.5 J·kg^−1^·K^−1^ across the intermediate region under the same field, ascribed to the presence of two types of manganites with different Curie temperatures. In addition, the maximum value of Δ*S*_M_ in the composite was lower than that in its individual manganite components. This reduction is characteristic of composite systems, where interphase interactions often moderate the peaks of Δ*S*_M_. Similar behavior is reported in composites of manganites and perovskites, where the peak shifts or diminishes due to phase mixing and distributed transitions. Maintaining stable and predictable Δ*S*_M_ over a broad temperature span is essential for reliable magnetocaloric performance [44,45,46,47,48,49,50,51,52].

The relative cooling power (RCP), a commonly used figure of merit for magnetocaloric materials and a key parameter for magnetocaloric applications, was calculated as the product of the maximum magnetic entropy change (${∆S}_{M}^{max})$ and the full width at half maximum ${\delta T}_{\mathrm{FWHM}}$ of the $\left|∆S_{M}^{max}\right|$ peak, following the equation below [4].

(5) $$\mathrm{RCP}=\left|{∆S}_{M}^{max}\right|\cdot {\delta T}_{\mathrm{FWHM}}$$

Therefore, the RCP depends not only on the peak of –Δ*S*_M_ but also on the width of the temperature interval over which significant entropy change occurs. The RCP values obtained for a magnetic applied field of 18 kOe are 55.24, 65.12, and 188.82 J·kg^−1^ for LCSM0.05, LCSM0.1, and the composite, respectively; these results confirm the increase in the RCP with the formation of the composite, as was predicted in this study. This reduction in the peak magnitude but extended operating temperature window (OTW) is attributed to the presence of multiple phases in the composite, where overlapping transitions broaden the Δ*S*_M_ profile and can increase RCP while lowering the peak of Δ*S*_M_. As shown in Table 3, which presents the magnetocaloric parameters of several lanthanum-based manganite composites, the material developed in this work exhibits one of the highest magnetic entropy changes (|ΔS_M_| = 5.86 J·kg^−1^·K^−1^) and a competitive relative cooling power (RCP = 188.8 J·kg^−1^) within an extended operational range (≈85 K around room temperature), confirming its superior magnetocaloric performance and the effectiveness of the dual-phase composite approach employed in this study.

A field normalization analysis was also performed (included in the Supplementary Materials), revealing that the field dependence of the magnetic entropy change follows the relation DS α H^n^. The exponent *n* was determined experimentally from the |ΔS_M_| data, obtaining values of 1.25 and 1.99 at 243 and 328 K, respectively, with an average value of n ≈ 1.65 near the transition temperature. Using this relationship, the DS_M_ values were normalized to standard magnetic fields of 2 T to facilitate a direct comparison with literature data. The maximum normalized values obtained were 6.7 and 2.8 J Kg^−1^ K^−1^ at 2 T, positioning this composite material among those with superior performance compared to previously reported systems. The quantified uncertainty ensures a reliable assessment of ΔS_M_ behavior and supports the consistency of the obtained results.

The improved magnetocaloric performance of the studied composite is attributed to interfacial exchange and strain-mediated coupling between the two manganite phases (compounds), which modify the local Mn-O-Mn geometry and broaden the magnetic transition. This coupling results in a non-additive RCP and smoother ΔS_M_, consistent with cooperative effects at the interfaces. However, further local analysis will be required to confirm this interpretation in future research.

Optimization strategies therefore must balance peak magnitude and the OTW (for example, via microstructure control, phase fraction tuning, or measurement at higher ΔH) to maximize usable refrigeration capacity for the target operating temperature range. Therefore, while composite formation led to enhancements in RCP and broadened the operating temperature window, the reduced peak values and lower intermediate temperatures pose a limitation. Optimization strategies, for example, stronger applied fields or improved composite architecture (phase proportions, microstructure), may mitigate these drawbacks and lead to materials with more uniform and elevated Δ*S*_M_ across the temperature range.

## 4. Conclusions

A composite powder formed by a mixture of La_0.7_Ca_0.2_Sr_0.1_MnO_3_ and La_0.7_Ca_0.25_Sr_0.05_MnO_3_ in an equal weight ratio was successfully obtained, with no evidence of secondary phases detected by X-ray diffraction and Rietveld refinement. The manganites were synthesized by the solid-state reaction route, using high-energy milling for 5 h and annealing at 1473 K for 2 h, while the composite was prepared by mixing both phases using high-energy ball milling for 2 min and applying a further heat treatment at 1473 K for 2 h. SEM and EDS analyses revealed a consolidated microstructure with good interfacial connectivity and controlled chemical heterogeneity, features that enhance the cooperative magnetic response and magnetocaloric performance of the composite. This approach confirms a simple and effective method for producing magnetocaloric composite ceramic materials. Magnetic analysis through Arrott plots shows that the composite undergoes a second-order phase transition according to Banerjee’s criterion, which is favorable for magnetic refrigeration since it minimizes magnetic and thermal hysteresis. Although the maximum |ΔS_M_| value of the composite is lower than that of the individual manganites, the relative cooling power reaches 188.82 J·kg^−1^, which is considerably higher than the 55.24 and 65.12 J·kg^−1^ obtained for LCSM0.05 and LCSM0.1, respectively. The non-additive RCP, the broader and smoother ΔS_M_, and EPR evidence confirm that the enhanced magnetocaloric response of the composite likely arises from interfacial exchange coupling and strain-mediated effects between the two manganite phases. This improvement points to a more efficient magnetocaloric performance of the composite, supporting its potential use in magnetic cooling applications. These results demonstrate the effectiveness of our synthesis route in enhancing magnetocaloric performance under practical, low-field conditions. Furthermore, considering practical aspects, the composite is expected to exhibit a moderate adiabatic temperature change (ΔT_ad_) consistent with its enhanced relative cooling power, negligible magnetic hysteresis losses due to its second-order transition, and good thermal and magnetic stability over repeated cycles, which are essential attributes for reliable magnetic refrigeration applications.

## Figures and Tables

**Table 1:** Phase quantification, lattice parameters, crystallite size (D), microstrain (µs), and goodness of fit obtained from Rietveld refinement of XRD patterns for LCSM 0.05, LCSM 0.1 and composite.

Sample	Phase/Space Group	Phase (wt. %)	D (nm)	Microstrain (Adim)	Lattice Parameter (Å)			Goodness of Fit	
					a	b	c	R_wp_	χ^2^
LCSM0.05	La_0.7_Ca_0.25_Sr_0.05_MnO_3_ *Pbnm*	100 ± 0.00	77.40 ± 4.26	4.76 × 10^−4^ ± 3.11 × 10^−6^	5.44 ± 6 × 10^−4^	7.76 ± 3 × 10^−4^	5.47 ± 6 × 10^−4^	20.89	1.18
LCSM0.1	La_0.7_Ca_0.2_Sr_0.1_MnO_3_ *Pbnm*	100 ± 0.00	79.01 ± 2.26	6.36 × 10^−4^ ± 3.72 × 10^−6^	5.48 ± 1 × 10^−4^	7.69 ± 1 × 10^−4^	5.46 ± 9 × 10^−4^	15.80	1.87
Composite	La_0.7_Ca_0.25_Sr_0.05_MnO_3_ *Pbnm*	50.40 ± 0.65	93.94 ± 5.46	1.77 × 10^−4^ ± 2.46 × 10^−6^	5.48 ± 5 × 10^−3^	7.72 ± 5 × 10^−3^	5.47 ± 5 × 10^−3^	21.26	2.47
	La_0.7_Ca_0.2_Sr_0.1_MnO_3_ *Pbnm*	49.60 ± 1.46	100 ± 5.50	6.09 × 10^−4^ ± 2.26 × 10^−6^	5.46 ± 4 × 10^−3^	7.71 ± 9 × 10^−3^	5.48 ± 7 × 10^−3^		

**Table 2:** Bond angles obtained using VESTA simulation data from XRD patterns.

Sample	Phase	Mn-O-Mn Bond Angle (Degree)	
		Mn_1_-O-Mn_1_	Mn_2_-O-Mn_2_
LCSM0.05	La_0.7_Ca_0.25_Sr_0.05_MnO_3_	160.29	161.32
LCSM0.1	La_0.7_Ca_0.2_Sr_0.1_MnO_3_	160.15	161.37
Composite	La_0.7_Ca_0.25_Sr_0.05_MnO_3_	160.19	161.36
	La_0.7_Ca_0.2_Sr_0.1_MnO_3_	160.24	161.36

**Table 3:** Comparative magnetocaloric parameters: Curie temperature (Tc), operating temperature window (OTW), magnetic entropy change (−∆S_M_), and relative cooling power (RCP) for different ceramic composites based on lanthanum manganites.

Composite	Tc (K)	${µ}_{0}H$ kOe	OTW (K)	$\left|{∆S}_{M}\right|$ (J·kg^−1^·K^−1^)	RCP (J·kg^−1^)	Reference
0.5La_0.75_Ca_0.25_Sr_0.05_MnO_3_/0.5La_0.7_Ca_0.2_Sr_0.1_MnO_3_	286–307	18	85	5.86	189	This work
0.5La_0.7_Ca_0.2_Sr_0.1_MnO_3_/0.5La_0.7_Te_0.3_MnO_3_	255–303	20	70	2.07	125	[7]
0.95La_0.45_Nd_0.25_Sr_0.3_MnO_3_/0.05CuO	285	15	95	2.55	75	[8]
0.4La_0.6_Ca_0.4_MnO_3_/0.6La_0.6_Sr_0.4_MnO_3_	255–350	30	250	0.92	215	[10]
0.5La_0.65_Ca_0.35_MnO_3_/0.5Pr_0.5_Sr_0.5_MnO_3_	277–298	20	100	0.68	80	[11]
0.75La_0.7_Ca_0.2_Sr_0.1_MnO_3_ /0.25La_0.7_Te_0.3_MnO_3_	290	20	70	2.5	138	[12]
0.5La_0.7_Ca_0.3_MnO_3_/0.5La_0.7_Ag_0.3_MnO_3_	260–304	20	98	2.65	139	[14]
0.75La_0.7_Ca_0.3_MnO_3_/0.25La_0.84_Sr_0.16_MnO_3_	255	20	140	0.72	89	[15]
0.5La_0.67_Ca_0.27_Sr_0.06_MnO_3_/0.5La_0.7_Sr_0.3_Mn_0.95_Cu_0.05_O_3_	264–360	20	75	1.82	138	[16]
0.5La_0.7_Ca_0.2_Sr_0.1_MnO_3_/0.5La_0.7_Ca_0.15_Sr_0.15_MnO_3_	310–336	20	90	1.26	115	[19]
0.33Pr_0.67_Ca_0.33_MnO_3_/0.66La_0.67_Sr_0.33_MnO_3_	346	20	250	0.98	77	[40]

## Data Availability

The raw and processed data required to reproduce these findings are available for download at https://data.mendeley.com/preview/3r8g4s3cr2?a=fbe060a9-6e85-454a-9eb1-0a728613bbb2 accessed on 17 October 2025.

## Supplementary Materials

The following supporting information can be downloaded at: https://www.mdpi.com/article/10.3390/ma18214869/s1, Figure S1: SEM-EDS; Figure S2: Normalized n; Figure S3: critical parameter; Table S1: g factor; Table S2: uncertainty.
