# Supplementary material for: An Enhancement in the Magnetocaloric Effect in a Composite Powder Based on Lanthanum Manganites
Source: Materials (Basel). 2025 Oct 24;18(21):4869. doi: 10.3390/ma18214869 (PMC12608266; doi:10.3390/ma18214869)
Supplement: Supplementary file 1 [file materials-18-04869-s001.zip › Table S1.pdf]

$g_{\text{eff}} = \frac{h\nu}{\mu_B H_{\text{res}}}$

LCSM0.05 at 293 k

$g = \frac{6.626\times10^{-34} J\cdot s * 9.64\times10^{9} GHz}{9.274\times10^{-24} J/T * 0.3458\text{T}}$=1.992

LCSM0.1 at 293 k

$g = \frac{6.626\times10^{-34} J\cdot s * 9.64\times10^{9} GHz}{9.274\times10^{-24} J/T * 0.3465\text{T}}$=1.988

Composite at 293 k

$g = \frac{6.626\times10^{-34} J\cdot s * 9.64\times10^{9} GHz}{9.274\times10^{-24} J/T * 0.3161\text{T}}$=2.179

LCSM0.05 at 378 k

$g = \frac{6.626\times10^{-34} J\cdot s * 9.64\times10^{9} GHz}{9.274\times10^{-24} J/T * 0.3473}$=1.983

LCSM0.1 at 378 k

$g = \frac{6.626\times10^{-34} J\cdot s * 9.64\times10^{9} GHz}{9.274\times10^{-24} J/T * 0.3447}$=1.998

Composite at 378 k

$g = \frac{6.626\times10^{-34} J\cdot s * 9.64\times10^{9} GHz}{9.274\times10^{-24} J/T * 0.3427}$=2.01
