# Supplementary material for: An Enhancement in the Magnetocaloric Effect in a Composite Powder Based on Lanthanum Manganites
Source: Materials (Basel). 2025 Oct 24;18(21):4869. doi: 10.3390/ma18214869 (PMC12608266; doi:10.3390/ma18214869)
Supplement: Supplementary file 1 [file materials-18-04869-s001.zip › Table S2.pdf]

Table. Determination of Uncertainty

Sample	Uncertainty ΔS_m ($J \cdot kg^{-1} \cdot K^{-1}$)
LCSM 0.05	±0.0145
Composite	±0.0192
LCSM 0.1	±0.0187
