# Supplementary figures and images for: An Enhancement in the Magnetocaloric Effect in a Composite Powder Based on Lanthanum Manganites
Source: Materials (Basel). 2025 Oct 24;18(21):4869. doi: 10.3390/ma18214869 (PMC12608266; doi:10.3390/ma18214869)

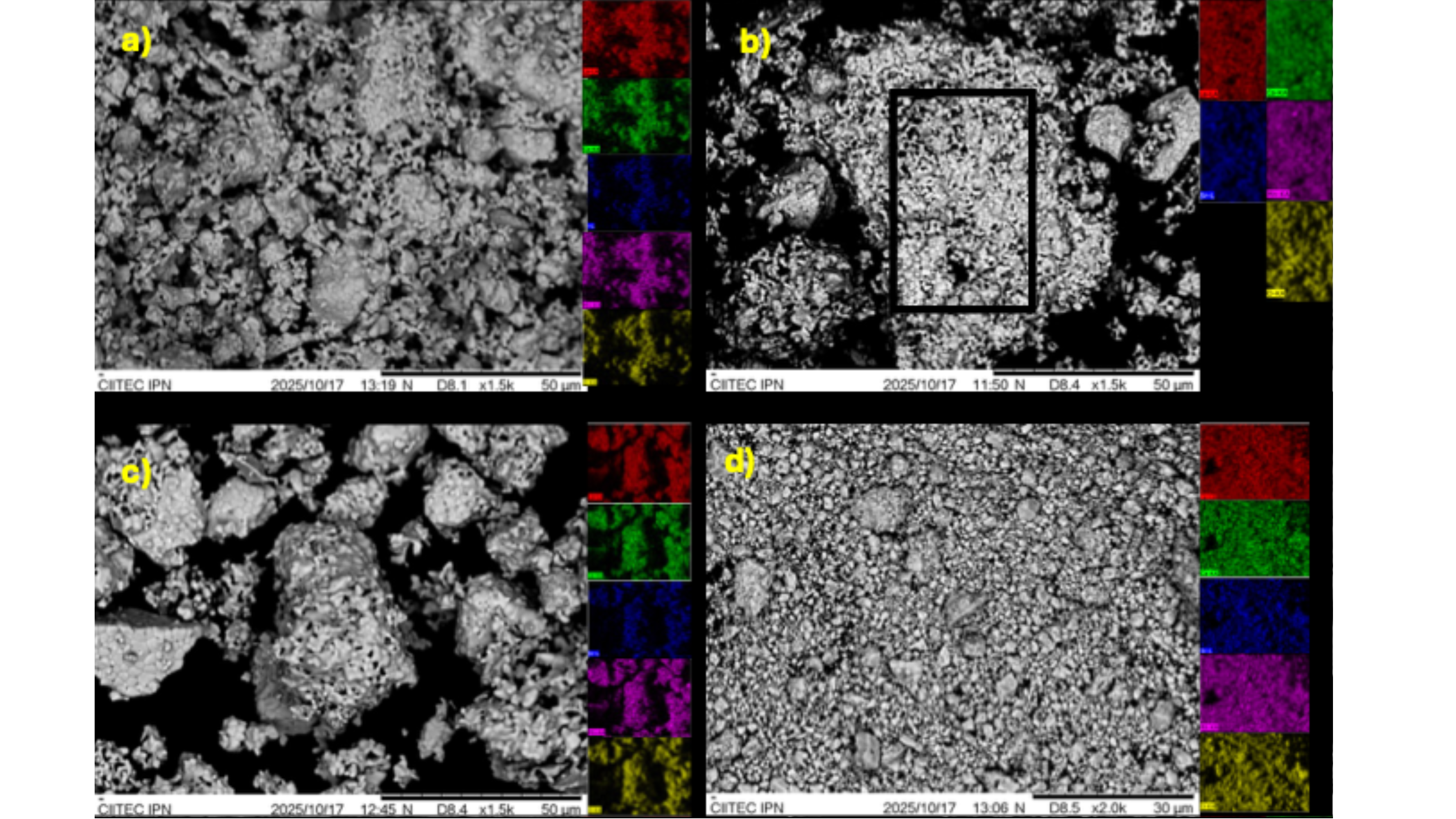

Supplement: Supplementary file 1 [file materials-18-04869-s001.zip › Figure S1.png]

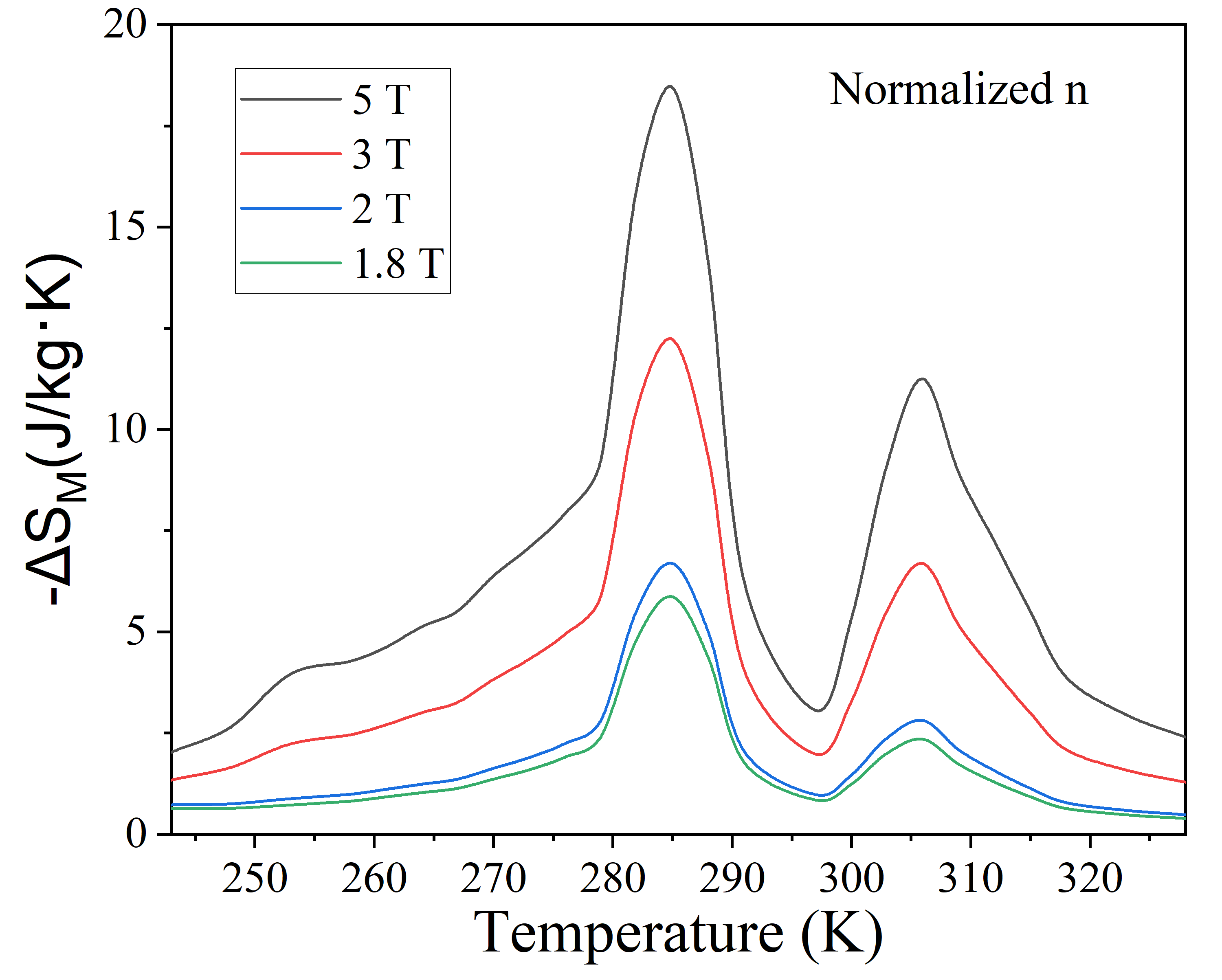

Supplement: Supplementary file 1 [file materials-18-04869-s001.zip › Figure S2.png]

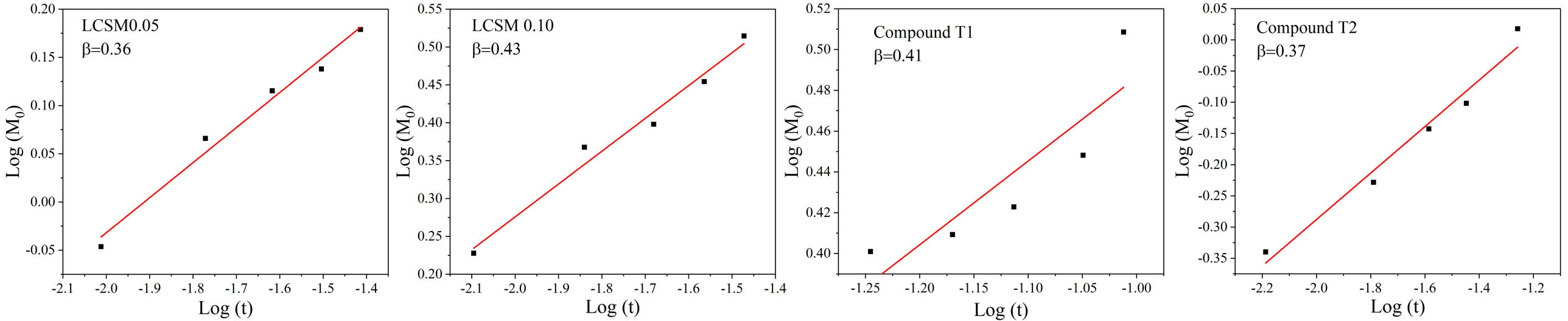

Supplement: Supplementary file 1 [file materials-18-04869-s001.zip › Figure S3.png]
